# Teaching prudent antibiotic use on the go: a descriptive report on development, utilization and listener satisfaction of an educational podcast format for medical students and young professionals

**DOI:** 10.1186/s13756-024-01402-8

**Published:** 2024-05-11

**Authors:** Sandra Schneider, Clara Bergmann, Felicia Becker, Lukas Risse, Caroline Isner, Hartmut Stocker, Markus A. Feufel, Alina Röhrig, Oliver Kurzai, Thiên-Trí Lâm, Stefan Hagel, Mathias W. Pletz, Petra Gastmeier, Miriam Wiese-Posselt

**Affiliations:** 1 Institute of Hygiene and Environmental Medicine, Charité – Universitätsmedizin Berlin corporate member of Freie Universität Berlin, Humboldt- Universität zu Berlin, Hindenburgdamm 27, Berlin, 12203 Germany; 2 Klinik für Innere Medizin-Infektiologie, Vivantes Auguste-Viktoria-Klinikum, Rubensstr. 125, Berlin, 12157 Germany; 3 Department of Infectious Diseases, St. Joseph Hospital, Berlin-Tempelhof, Wüsthoffstr. 15, Berlin, 12101 Germany; 4 Department of Psychology and Ergonomics (IPA), Division of Ergonomics, Technische Universität Berlin, Marchstr. 23, Berlin, 10587 Germany; 5 Institute for Hygiene and Microbiology, University of Würzburg, Josef-Schneider-Str. 2, Würzburg, 97080 Germany; 6 Institute for Infectious Diseases and Infection Control, Jena University Hospital, Am Klinikum 1, Jena, 07747 Germany; 7 Helios Klinikum Emil-von-Behring, Walterhöfer Str. 11, Berlin, 14165 Germany

**Keywords:** Antibiotic stewardship, Antimicrobial stewardship, Medical education, FOAM, Antimicrobial resistance, Podcast

## Abstract

**Background:**

An important component in fostering the responsible use of antibiotics is training of new and future prescribers in this interdisciplinary topic. Because podcasts are playing an increasing role in medical education, we aimed to develop and evaluate a podcast format with practice and guideline-oriented learning content on antibiotic therapy for medical students and young medical professionals.

**Methods:**

We developed the concept for the podcast with the direct involvement of medical students and medical experts with teaching experience. We used video conferencing when recording the episodes in order to have quick, easy, and nationwide access to the experts involved. We released an episode every 2 to 4 weeks on the popular podcast platforms. The podcast was promoted through mailing lists, social and print media, and at conferences. The evaluation of episodes was based on user data provided by the platforms and an anonymous feedback questionnaire linked to each episode in the podcast notes.

**Results:**

Between December 2021 and December 2022 19 episodes of *InfectEd: der Antibiotika-Podcast* were released. The mean duration of an episode was 91 min. By March 9, 2023, a total of 38,829 downloads and streams had been recorded. The majority of users listened to the podcast on a mobile device. The average playing time per episode was 65%. The feedback questionnaire was completed 135 times. 60.7% of respondents were female, 38.5% male. The majority of respondents were in their twenties and thirties (66.7%). 31.1% were medical students, 25.9% were residents, and 25.2% were specialists. Listeners were asked to rate episodes on a scale from 1 to 6, where 1 was “very good” and 6 was “insufficient.” Ratings did not differ significantly between female and male respondents or between medical students and others. 118 respondents (87.4%) reported an increase in knowledge. Free-text feedback frequently emphasized clinical and also exam relevance.

**Conclusion:**

Our podcast format, developed with a user-centered approach, was broadly distributed and has been well accepted by both medical students and physicians alike. It provides a large number of learners with low-threshold access to current, guideline-orientated content and could be a useful supplement to conventional teaching formats.

**Supplementary Information:**

The online version contains supplementary material available at 10.1186/s13756-024-01402-8.

## Background

Increasing antimicrobial resistance (AMR) is a major challenge in modern medicine, and the misuse and overuse of antibiotics is an important driver in the development of AMR [1]. Medical students as future prescribers and young physicians as new prescribers should receive in-depth training in responsible and guideline-adherent antibiotic use. The InfectControl Project RAI students (RAI = **R**esponsible **A**ntibiotic use via **I**nformation and Communication, target group medical students) addressed the question how modern learning concepts can be designed to communicate the complex and interdisciplinary issues of antibiotic therapy to medical students [2, 3].

The project has resulted in four digital teaching formats, each free of charge, each addressing different learning preferences and knowledge levels: (a) Explanatory films that provide short pieces of information; (b) a structured online course (“massive open online course” = MOOC) that teaches microbiological and pharmacological basics and treatment principles; (c) interactive case studies that serve to train practical knowledge; finally, (d) the podcast format to teach concrete antibiotic therapy decision-making based on a deep understanding of the respective clinical picture and target-oriented diagnostics. Hence, its primary addressees are advanced medical students and young physicians.

Internationally, podcasts are already in wide use in medical education [4–6]. A podcast format on infectious diseases with six tutorial episodes was evaluated at the University of London in 2011 and rated “excellent” by a majority of the London medical students surveyed [7]. However, it is not easy to transfer learning content on guideline-based antibiotic therapy internationally—in particular because of regional differences in antibiotic resistance and differences of national guidelines from country to country. This implies a need for content that is aligned to relevant national guidelines. Another challenge in developing an open-access learning format for medical students and young physicians is to reach audiences with widely varying levels of prior knowledge while remaining clinically relevant.

This article aims to describe the user-centered development of our German podcast format and to assess its reception on the base of usage data and a voluntary feedback questionnaire.

## Methods

### Study design and research questions

The study was designed as a feasibility study with a pragmatic approach. The question was how our podcast format on the practical use of antibiotics was received by the target group in terms of acceptance, accessibility, utilization, and satisfaction.

### Conception

The actual concept development of the podcast was preceded by an analysis of the target group. This involved a professional service provider (Point Blank – Research & Consultancy GmbH, Berlin), which conducted interviews with young physicians as well as focus group discussions with medical students and lecturers from various fields. We also conducted an online survey on knowledge, attitudes, and behavior (KAB survey) regarding AMR and antibiotic use as well as the learning preferences of medical students regarding access, format, and content [2, 3].

In this first project phase, which preceded this work, we learned that in addition to gaps in knowledge regarding the use of antibiotics, there is above all a lack of practical competence in the student population and among young physicians, which results in a demand for strongly practice-oriented knowledge transfer.

We also learned that the individual level of knowledge and skills in dealing with antibiotics varies greatly in these groups. The focus groups revealed that there are few preferences in terms of format or medium as long as the content is clinically relevant. In the end, we chose the podcast format because in our opinion it enables quick digital access to concrete practical knowledge, conveyed by experts and peers in sufficient depth.

In addition to the preceding project phase, we incorporated the experience gained from a newly designed Charité elective module for medical students on antibiotic therapy.

Medical students were then continuously involved in the development of the podcast in order to get constant feedback from the target group in the sense of participatory design.

### Development and production

Topics for podcast episodes were determined by the RAI students study group based on previous clinical and teaching experience. In addition, feedback from the listeners was incorporated into the selection of topics for later episodes.

Each podcast episode consisted of questions from two medical students on a particular infectious disease topic and the answers of a clinical expert. A physician from our study group acted as moderator after an external moderation training. The language of the podcast was German. Each sequence was broadly structured in advance in topic blocks such as the clinical picture, risk factors, diagnostics, and therapy. Beyond that, there was no script because we were striving for an authentic discussion atmosphere. Each episode lasted between 1 and 2 h. The recording sessions took place via video conference. Recording and post production were performed by a professional producer (Karsten Kretzer, Kretzer TV, Berlin). The cover was designed by a professional graphic designer (Steffen Kalauch – Visuelle Kommunikation, Berlin).

### Distribution

The podcast’s Internet host was Podigee (www.podigee.com), which published the episodes on its own platform as well as on other popular podcast platforms, such as Apple, Spotify, etc. Advertising was conducted via email to student representatives and lecturers at all German-speaking medical faculties and via the RAI Twitter account [8]. A new episode was released every 2 to 4 weeks, always on the same day of the week. In addition, the podcast was linked in the RAI-students’ YouTube channel of explanatory films [9] and in the MOOC [10]. In June 2022, the podcast was promoted on a general German medical podcast with a broad distribution [11]. In September 2022 the podcast was presented at the annual meeting of the German Society for Hygiene and Microbiology along with the other formats developed in the RAI students project. Starting in September 2022, stickers, flyers, and posters were distributed at various scientific events.

### Evaluation and analysis

To analyze utilization and accessibility we used standard usage data collected by the provider platform Podigee and Apple Podcast. To assess acceptance and satisfaction we used a voluntary 9-item feed back questionnaire with closed and open questions.

Data on downloads and streams (D&S) and listeners were made available in aggregated form by Podigee. According to Podigee, listeners were identified by IP address and user agent. D&S raw data was cleaned up by a 5-step filtering and interpretation process prior to data transmission. Only D&S which lasted at least 1 min were counted, and multiple D&S by the same listener within a 24-hour period were aggregated into a single D&S [12].

Since Podigee cannot access information on playing time, this information was retrieved from Apple Podcasts Connect for the users of the Apple Podcast platform. We did not have access to this data for other platforms.

A voluntary anonymous feedback questionnaire was linked in the show notes of each episode. Completing the questionnaire was promoted in each podcast episode’s outro. The sampling method followed a convenience strategy. The 9-item questionnaire was programmed using LimeSurvey software (including 3 open questions). A complete list of questions can be found in Supplement 1.

All data was extracted on March 9, 2023.

The data collected by the closed questions was analyzed descriptively using IBM SPSS. P-values were calculated by the two-sided Fisher’s Exact Test or Chi Square test where appropriate. Tests were regarded as significant for *p* ≤ 0.05. An orientating qualitative analysis of the answers to the open questions was performed without special software. Categories were created manually after reading through the free text comments.

## Results

### Usage profiles

Between December 2021 and December 2022, a trailer, 19 episodes and one brief organizational information message were released under the label *InfectEd: der Antibiotika-Podcast* [13]. By March 9, 2023, a total of 38,829 D&S had been registered. On average, each episode was played 1859 times. Table 1 shows the number of D&S by episode. The podcast was most frequently accessed on smartphones (86.0% of D&S). The most common platforms used were Spotify (55.1% of D&S) and Apple Podcast (28.7% of D&S). On the Apple Podcast platform, the average episode playing time was 65% of the respective episode length.

**Fig. 1:**
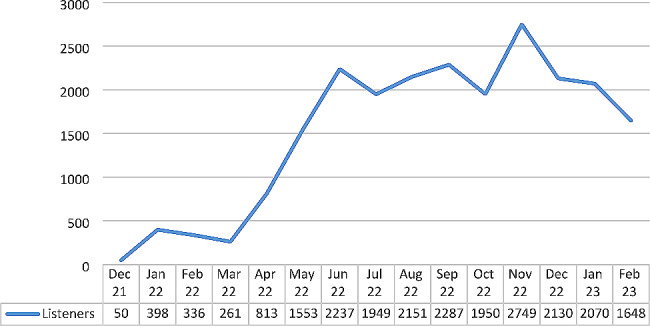
Number of podcast listeners over time

The number of podcast listeners (as defined by Podigee) started at 50 after the release of the first episode in December 2021 and peaked in November 2022 at 2749. Even after the release of the last episode in December 2022, the number of listeners remained high in January and February 23 (Fig. 1).

**Table 1:** Aired episodes of “InfectEd – der Antibiotika-Podcast”

Episode No.	Topic	Release date	Duration (minutes)	Downloads and streams *n*	Completed Questionnaires *n*
X	Trailer	20-Dec-21	3	1027	xx
1	Community acquired pneumonia	21-Dec-21	95	5354	18
2	*Staphylococcus aureus* bacteremia	4-Jan-22	93	3966	20
3	*C difficile* infection	18-Jan-22	94	2531	9
4	Skin and soft tissue infections	1-Feb-22	100	2245	16
5	Infection prevention and hospital hygiene	15-Feb-23	108	1766	3
XX	Organizational information	15-Mar-22	2	628	xx
6	Microbiological diagnostic	29-Mar-22	101	1985	6
7	Opportunistic infections in HIV	3-May-22	95	1394	2
8	Perioperative antibiotic prophylaxis	31-May-22	72	1370	6
9	Tonsillitis and otitis media in childhood	21-Jun-22	103	1700	5
10	Invasive fungal infections	12-Jul-22	111	1520	8
11	Exacerbated COPD	26-Jul-22	68	1399	5
12	Pharmacological aspects of antibiotics	9-Aug-22	100	2000	9
13	One Health	23-Aug-22	73	1073	3
14	Endocarditis	6-Sep-22	78	1309	5
15a	Urinary tract infections – diagnostic	20-Sep-22	74	1142	5
15b	Urinary tract infections – therapy	21-Sep-22	50	1154	
16	Hospital acquired pneumonia	18-Oct-22	87	1194	5
17	Sepsis	1-Nov-22	79	1303	6
18	Vaccination	15-Nov-22	123	1174	2
19	Tuberculosis	13-Dec-22	106	1596	2

### Description of the respondents to the feedback questionnaire and podcast rating

The feedback questionnaire was completed a total of 135 times. For the number of questionnaires completed per episode, see Table 1.

**Fig. 2:**
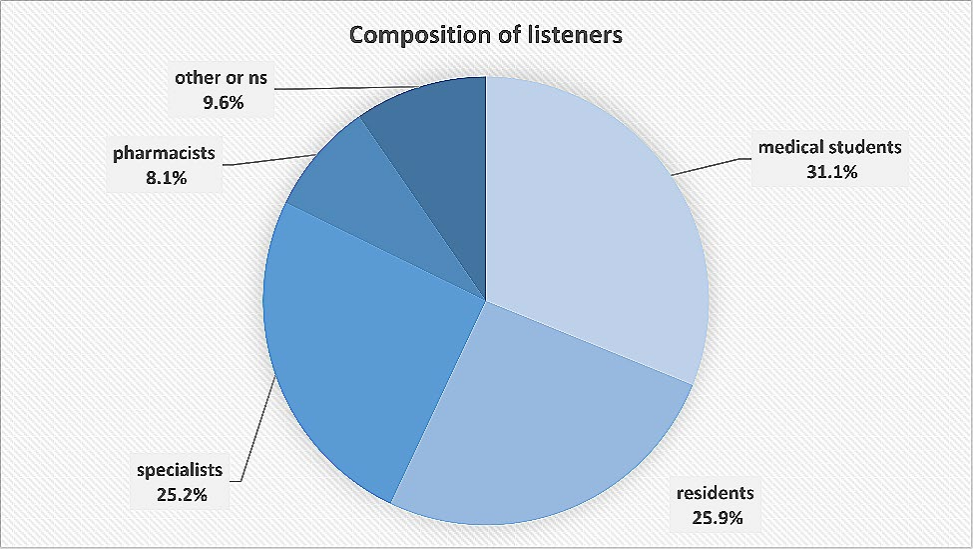
Composition of listeners who responded to the questionnaire linked in the show notes of the podcast episodes; ns = not specified

60.7% of respondents were female. The majority of respondents were in their twenties (40.0%) and thirties (26.7%). 31.1% (*n* = 42) were medical students (Fig. 2). Of these, 17 were in semester 1 to 6 and 17 in semester 7 to 9 and 8 without semester indication (the ninth semester had the highest number of listeners). The following study locations were indicated: Berlin, Bochum, Dresden, Düsseldorf, Erlangen, Freiburg, Göttingen, Greifswald, Heidelberg, Homburg-Saar, Jena, Kiel, Lübeck, Mainz, Munich, Nuremberg, Tübingen, Würzburg, and Vienna. This accounts for about half of all the public medical schools in Germany together with an Austrian university. For further information on the non-medical students, see Fig. 2. For full description of respondent characteristics, refer to supplement 1.

**Fig. 3:**
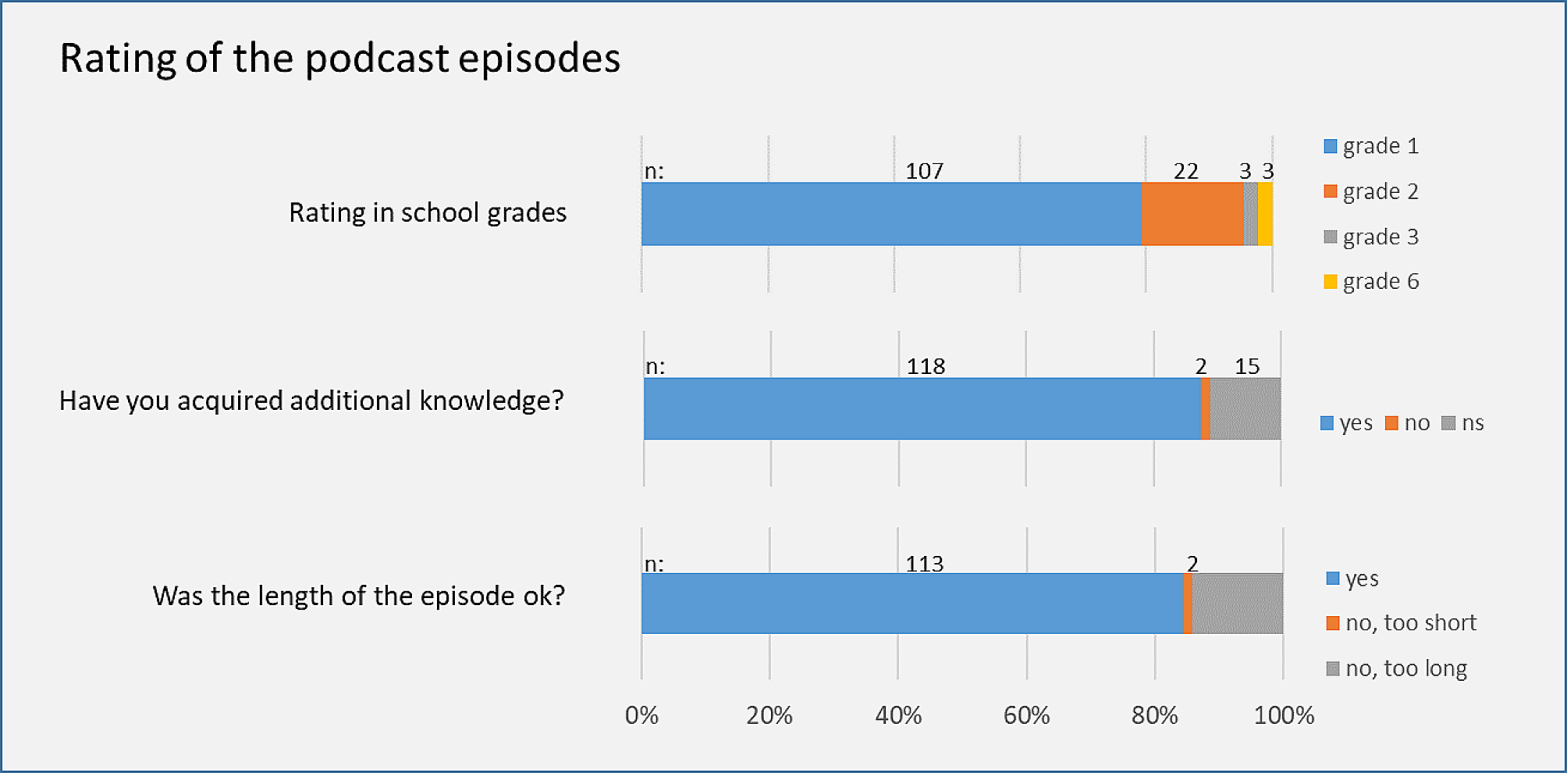
Rating of the podcast episodes by the questionnaire linked in the show notes: global rating on a scale from 1 to 6 (German school grades) with 1 = very good, 2 = good, 3 = satisfactory, 4 = sufficient, 5 = poor, 6 = insufficient; ns = not specified

Figure 3 shows the quantitative rating of the podcast episode.

On a scale from 1 to 6 (1 = very good; 6 = insufficient), grade 1, the highest grade, was awarded by 79.3% of all respondents. The average grade was 1.32. Grade assignment did not differ significantly between female and male respondents, or between medical students and others. It did differ by age group however (*p* = 0.01). Thus, the group of 30 to 39-year-olds awarded the grade 1 most frequently (86.1% of the age group).

A knowledge gain was reported by 87.4% of all respondents. Medical students were significantly more likely to report a gain than other respondents: 97.6% of students but only 82.8% of other respondents reported a knowledge gain (*p* = 0.02).

When asked if the length of an episode was appropriate, 83.7% of all respondents answered “yes”. Medical students were more likely to approve of the length of an episode than other participants: 92.9% of students but only 79.6% of other participants found episode length appropriate (*p* = 0.08). For full description of quantitative feedback refer to supplement 1.

### Informal feedback

**Fig. 4:**
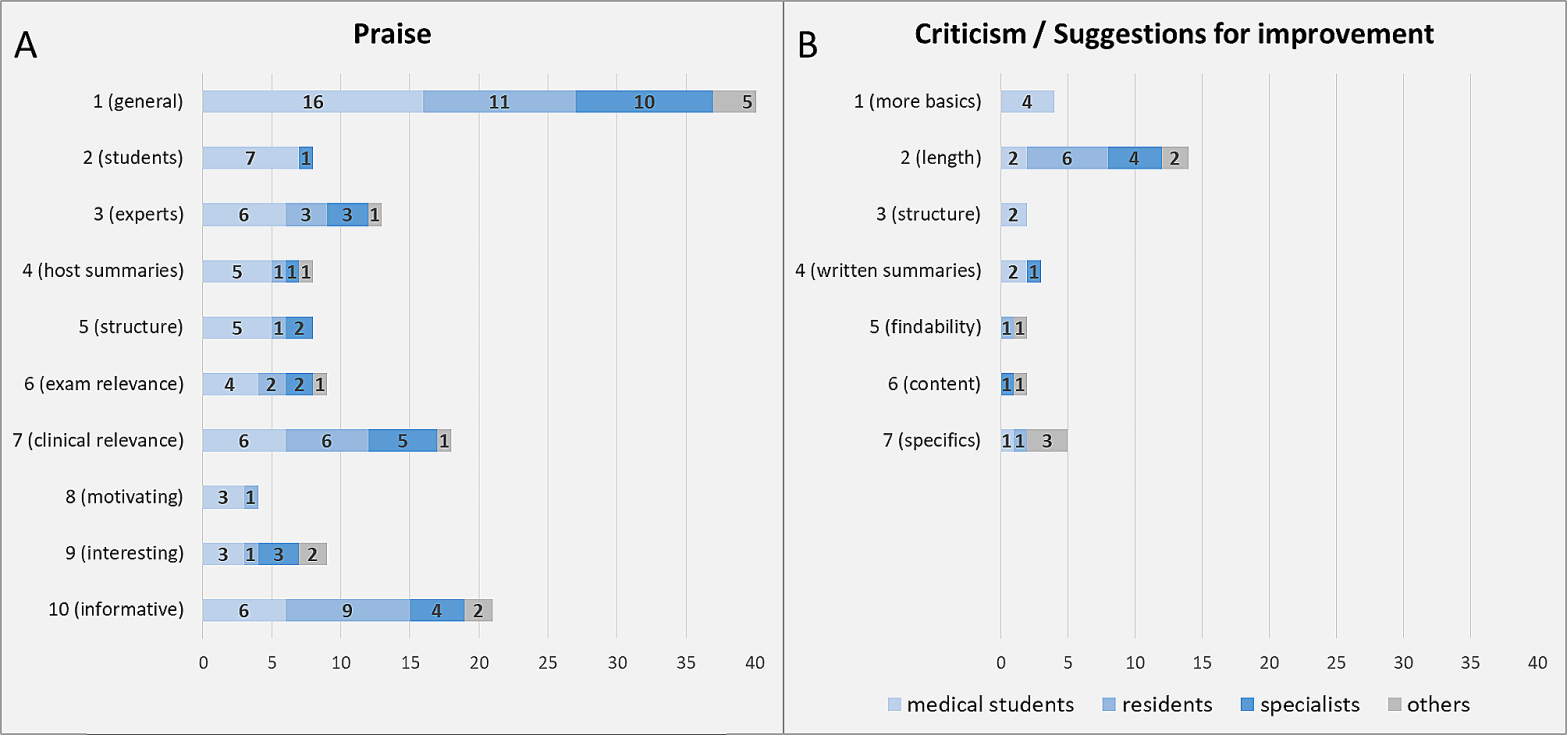
Theme clusters of the text fields for praise and criticism/ suggestions for improvement in the questionnaire linked in the each podcast episode’s show notes. (See main text for details on the cluster categories)

Regarding the open question fields in the questionnaire: 56.3% entered a comment in the free text field for positive feedback. An orientating qualitative analysis of the praise texts identified 10 recurring categories: (1) general praise for the format, *e.g. “great podcast” or “very good format” or “I am totally exited about the episodes”;* (2) praise for student participation, *e.g. “good with the students asking the questions” or “good concept: medical students were there and asked important questions”;* (3) praise for the experts, *e.g. “the lecturer explained everything very well” or “top notch experts”;* (4) praise for the summaries by the host, *e.g. “Moderator/physician summaries, they really help!” or “interim conclusions of the moderator – the best”*; (5) praise for the structure, *e.g. “well structured” or “working through the clinical picture, diagnostics and therapy in a structured way”;* (6) emphasis on relevance for exams or didactic value, *e.g. “interesting supplement to regular teaching” or “great for learning” or “incredibly helpful for everyday life and preparation for my exam in microbiology”;* (7) emphasis on clinical relevance, *e.g. “Pragmatic and practice-relevant content for everyday clinical work.” or “Absolutely to be recommended, even for experienced clinicians.”;* (8) describing the episode as motivational or the like, *e.g. “Motivates me to look at the guideline.” or “Learning is so much fun: )”;* (9) describing the episode as interesting, exciting or the like, *e.g. “very interesting” or “totally exciting and entertaining”;* (10) describing the episode as informative or the like, *e.g. “very informative” or “Took a lot of notes for my daily routine”*. Most feedback addressed several of these categories. Only a single response in the positive feedback field was actually a criticism. Figure 4A shows the number of comments broken down by category and level of training. Table 2 shows translations of selected original comments.

**Table 2:** Selected translations of original comments in the praise text field. For the entirety of all free-text translations (praise and criticism suggestions for improvement, see Supplement 1)

“Interesting supplement to regular teaching, the questions from the student perspective are especially nice, those are the questions you ask yourself too.”
“Super explained! What is otherwise dry and rather unpopular, is worked up with so much care here that the interest is contagious! I think it is especially good that in between things are summarized and recapitulated.”
“I am totally excited about the episodes! I feel like I’m really learning an insane amount by working through the clinical picture, diagnostics and therapy in a structured way. Please keep up the good work! Learning is so much fun: )”
“I have been working as a nephrology resident in Austria for 3 years now and I learn lots of new things from every episode. In some cases, knowledge that was lost in clinical practice is revived, so that you can improve the way of you work. I am generally a fan of medical podcasts, unfortunately there are very few good ones from German-speaking countries and medical practice is sometimes quite different from the US. This podcast is definitely recommendable!”
“Really really great format in this episode!! I listened to the episode 2 times and even took notes the second time. So much information in one podcast! Really great - thank you and keep up the good work!! :)”
“Very detailed, great effort to provide background information and evidence for recommendations, repetition of key facts & recommendations for successful learning, top-notch experts”

26.7% of respondents entered comments in the free text field for criticism or suggestions for areas for improvement. An orientating qualitative analysis revealed that 6 of the 36 entries were actually further positive feedback. The 30 remaining statements could be roughly assigned to 7 categories: (1) a desire for more basic information, *e.g. “Repeat basic knowledge a little more” or “lead-in to AB therapy too fast and section about AB too short, I would have liked more repetition of the content”;* (2) criticism of the length of the episode or specific parts of the episode, *e.g. „If it were a bit shorter, it would be easier to listen to the whole thing in one sitting.” or “too long”;* (3) a desire for more structure, *e.g. „A bit more structure sometimes wouldn’t hurt”;* (4) a desire for written summaries, *e.g. “A summary or key aspects in the show-notes would be the greatest!!!” or “Script of the episode, if possible?”;* (5) the findability of the podcast on the Internet, *e.g. “you can’t find it”;* (6) criticism of specific statements made during the episode, *e.g. “Doubling the amount in the directions for use as a recommended dose in general in the case of sepsis is negligent in my opinion!!!”;* (7) very specific individual aspects, *e.g. “I would be happy if every now and then the work of the technical assistants were mentioned.”*. Overall, the criticism was relatively well-intended and was usually given together with positive feedback. Only 3 comments expressed severe criticism, none of which were from medical students. Figure 4B shows the number of comments made by category and level of training.

The frequency of qualitative feedback did not differ significantly between women and men, age groups, or medical students and other respondents.

For full description of qualitative feedback see supplement 1.

## Discussion

In this article, we present the development and evaluation of a German-language training podcast format concerning prudent antibiotic therapy. To our knowledge, there is only one podcast in the German-speaking world, *Antibiotic Stewardship* [14] (with 5 episodes), that also deals explicitly with this topic. Another German-language format (*Infektiopod* [15]) also frequently deals with antibiotic therapy as part of its infectious disease profile. Unlike ours, the primary intended audience of both these formats tends to be physicians who have been in clinical practice for a longer period of time. Thus, our format *InfectEd - der Antibiotika-Podcast* is unique in the German-speaking world.

User data from podcasts should be interpreted with caution since conclusions about individual listeners based on D&S, IP address and user agent are imprecise, and little can be said about listening behavior of users. Caution must also be exercised when comparing user figures from different platforms, since they might utilize different counting criteria. Nevertheless, routinely collected data provides some clues. We can see that it took some time for our format to achieve a certain level of awareness, but once it did it remained relatively stable from June 2022 onward with more than 2000 estimated listeners per month. The podcast was still being listened to in high numbers during the 2 months after the last episode was released. An interesting bit of additional information is the playing time data from the Apple platform (Apple Podcasts Connect), which shows that the average listening time is high. We didn’t have the playing time data for other platforms, but Apple Podcast accounts for nearly one-third of our D&S. This leads us to assume that users maintain interest during podcast episodes.

The feedback questionnaire was completed by only a few in relation to the total estimated number of listeners. This may be partially due to the fact that filling in the questionnaire on a mobile phone—which served as the medium for most listeners—can be somewhat long and tedious. Nonetheless, we received 135 responses that can be evaluated. It should be noted that the number of discrete individuals may be lower since some listeners probably rated several episodes. The results are therefore not to be regarded as representative but rather as orientational. Nevertheless, they provide interesting insights: Most respondents were from the medical field and a few were from related professions. Thus, taken as a group, the respondents correspond to our intended audience. In addition to the primary target group of medical students and young physicians (residents), it is noteworthy how many medical specialists responded. And even though more medical students than physicians reported an increase in knowledge, the general assessment of the format did not differ between the groups. This suggests that a format involving medical students and experts can appeal to a medical audience with a wide range of prior knowledge. However, it should be added that the few very critical comments made in the free-text fields came mainly from specialists and nonmedical respondents (data link of individual citations and professional status not shown). Although this does not comprise a great deal of feedback overall, it suggests that the format may, after all, be best suited for the primary intended audience of medical students and young residents.

An important goal of our concept was to combine basic understanding with a high degree of clinical relevance. But the contents taught should also have relevance for medical school exams. Therefore, it was encouraging that we received praise in the free texts for clinical relevance 18 times including comments from specialists and 9 times for exam relevance/didactic value, while at the same time a desire for more basic information was expressed only 4 times.

Podcasts are assuming an increasing role in medicine and science around the world. Some authors predict that an important place in medical education for them is inevitable [16]. There are many different podcast formats to choose from, just as in non-medical podcasts: informal conversations, didactically scripted episodes, expert interviews, literature reviews, storytelling, case histories, etc [17]. . . We opted for a more informal concept in which medical students interviewed a designated expert. We usually focused on one specific bacteriological condition per episode, its causes, diagnosis, differential diagnoses, and therapy. Some structure was added by the moderator. This format had the following advantages: the content became very practice-oriented. The student audience was represented by peers and if something was not understood, they could ask questions about it. Determining subtopics in advance along with the degree of clinical and didactic experience on the part of the experts allowed us to discuss all essential aspects of the respective topic although no script was used. Remote recording enabled experts nationwide to take part despite their tight schedules. The conversational atmosphere helped teach not only medical content but also soft skills and attitudes.

One disadvantage of the format was the length of episodes. While there are no studies that proof an optimal episode length, there is some evidence that episodes under 20 min in length are preferred [17, 18]. Our episodes lasted 60 to 120 min. The playing time data and the 83.7% agreement that the length was appropriate suggest that the high information content in our format favored the podcasts’ length. This shows that episode length is only one of many criteria and that there is no “single recipe” for a successful medical podcast. When answering the closed question, physicians found the episodes too long more often than medical students. The length was also the most frequently mentioned point of criticism in the free texts (14 mentions altogether but only 2 by medical students). In some cases, specific suggestions were made for shorter intros or rounds of introductions, so that time savings could be made there. A supplementary written summary was requested several times. This could be a good addition to long podcast episodes.

One advantage of the RAI students project [3] is certainly that the podcast fits into complementary e-learning formats, so that by combining formats different learning types and learning qualities can be addressed. This corresponds to the call from experts in medical education for an adaptive curriculum that allows for individualized learning strategies [19].

The following limitations of our descriptive report should be taken into account:

- An analysis of user data based on downloads and streams can only provide a rough estimate of the number of listeners and no insight into individual listening behavior. Information on listening time is only available for Apple Podcast users.
- Due to the open distribution of the podcast on all common platforms, there is no clearly defined study group and the sampling of the feedback questionnaire did not follow a structured sampling design, but rather a pragmatic approach.
- It can be assumed that there is a sampling bias and that listeners who found the podcast particularly good or particularly bad responded. No generalization can be made for the entire audience.
- The same listeners may have completed several feedback forms for different episodes, so that the number of respondents is lower than the number of questionnaires.
- Our findings can provide an orienting insight into the reception of our podcast format. However, it is not possible to make a quantitative statement about how many students and young professionals would use this format instead of or in addition to other formats.
- We did not investigate whether our podcast had a learning effect on listeners. It was only possible to describe individual statements that the episodes were perceived as informative and were sometimes used for exam preparation.

Despite these limitations, we think that our findings can encourage other (university) teachers to consider a podcast format as a supplementary teaching tool, preferably with the involvement of students.

Further didactic knowledge could be gained in future studies by creating laboratory conditions. For example, podcast formats with and without student participation or shorter versus longer episodes could be tested with regard to learning success.

## Conclusions

Our medical podcast format that focuses on bacterial diseases and antibiotic therapy has attracted widespread interest in the professional medical community in Germany, from medical students to specialists. Involving students in the expert interview format also makes it possible to address basic questions. This gives students the feeling that they are represented. Advanced listeners are confronted again with questions they might not ask themselves during their everyday routine. The format provides a large number of learners with low-threshold access to current, guideline-compliant content delivered by high-profile, national experts from a respective area. This knowledge can be picked up while tending to other activities (on the way to work, while doing housework, while exercising). Compared to monologue teaching formats, a podcast dialogue can explore a clinical topic from diverse perspectives. Taken together, a good podcast format can be a useful supplement to textbooks, commercial platforms, and the local university curriculum.

### Electronic supplementary material

Below is the link to the electronic supplementary material.

Supplementary Material 1

Supplementary Material 2

## Data Availability

Regarding D&S as well as playing time, we used the aggregate data routinely provided to podcast publishers by Podigee and Apple. These are transmitted continuously. Screenshots from the time point of analysis are provided in the supplement (Supplement 2). Raw data was not available to us at any time. Regarding the feedback questionnaire data a full description of all answers is provided in the supplement (Supplement 1). The raw dataset is available from the corresponding author on reasonable request.

## Abbreviations

AMR: antimicrobial resistance
D&S: downloads and streams
KAB: knowledge, attitudes and behavior
MOOC: massive open online course
n: count
No.: sequential number
Ns: not specified
RAI: responsible antibiotic use via information and communication (project name)
