# Supplementary material for: Teaching prudent antibiotic use on the go: a descriptive report on development, utilization and listener satisfaction of an educational podcast format for medical students and young professionals
Source: Antimicrob Resist Infect Control. 2024 May 11;13:50. doi: 10.1186/s13756-024-01402-8 (PMC11088774; doi:10.1186/s13756-024-01402-8)
Supplement: Supplementary file 1 — Supplementary Material 1 [file 13756_2024_1402_MOESM1_ESM.docx]

**Supplement 1**

**Descriptive analysis of feedback questionnaire**

**1. How did you like the episode you just listened to? (Rate it based on German school grades)**

*Wie hat Ihnen die Folge, die Sie gerade gehört haben, gefallen? (Bewerte anhand von Schulnoten)*

|  | **Number** | **Percent** |
| --- | --- | --- |
| **1 = „sehr gut“ ≈ American A** | 107 | 79.3 |
| **2 = „gut“ ≈ American B** | 22 | 16.3 |
| **3 = „befriedigend“ ≈ American C** | 3 | 2.2 |
| **4 = „ausreichend“ ≈ American D** | 0 | 0 |
| **5 = „mangelhaft“ ≈ American F** | 0 | 0 |
| **6 = „ungenügend“ ≈ American F** | 3 | 2.2 |
| **Total** | 135 | 100.0 |

**2. Did you learn anything new?**

*Haben Sie inhaltlich etwas dazu gelernt?*

|  | **Number** | | | **Percent** |
| --- | --- | --- | --- | --- |
| **Yes** | | 118 | 87.4 | |
| **ns** | | 15 | 11.1 | |
| **No** | | 2 | 1.5 | |
| **Total** | | 135 | 100.0 | |

**3. Was the length of the episode OK?**

*War die Länge der Folge ok?*

|  | **Number** | **Percent** |
| --- | --- | --- |
| **ns** | 1 | 0.7 |
| **Yes** | 113 | 83.7 |
| **No, too short** | 2 | 1.5 |
| **No, too long** | 19 | 14.1 |
| **Total** | 135 | 100.0 |

| **4. Give praise here:**  *Hier ist Platz für Lob:* | **5. Give criticism or suggestions for improvement here**  *Hier ist Platz für Kritik oder Verbesserungsvorschläge* |
| --- | --- |
| *Original German comment*  English translation | |
| *super Podcast, gut mit den Studentinnen, die die Zwischenfragen stellen, guter Überblick über klinische Aspekte*  great podcast, good with the students asking the questions, good overview of clinical aspects | *zu schneller Einstieg in die AB-Therapie und zu kurzer Abschnitt über AB, da hätte ich mir mehr Wiederholungen des Inhalts gewünscht*  lead-in to AB therapy too fast and section about AB too short, I would have liked more repetition of the content |
| *Sehr schön und strukturiert! Habe mir viele Notizen gemacht für den Alltag!  (Assistenzarzt Innere im 1. Jahr)*  Very nice and well-structured! Took a lot of notes for my daily routine!  (Resident Internal Medicine in the 1st year) | *Weiter so!*  Keep it up! |
| *Vielen Dank, schöner Podcast mit guten Fragen und tollem Experten!*  Thanks a lot, nice podcast with good questions and great experts! |  |
| *Bin schon lange als Arzt tätig, aber hätte mir solche Fortbildungen auch früher gewünscht. Hatte heute profitiert. Vielen Dank*  Have been a physician for a while now, but would have liked to have had such a course earlier. It helped me today. Thanks a lot |  |
| *Gute Mischung aus Basic-Informationen und notwendigem Tiefgang, so dass für jeden etwas neues dabei ist.*  Good mix of basic information and necessary depth, so there is something new for everyone. |  |
| *Ich finde die Folge sehr sehr gut. Die Fragen waren sehr gut und die/der Dozent*in hat sehr gut alles erklärt.*  I think the episode was very, very good. The questions were very good and the lecturer explained everything very well. | *Wenn es bisschen kürzer wäre, könnte man das einfacher am Stück hören.*  If it were a bit shorter, it would be easier to listen to the whole thing in one sitting. |
| *- gutes Format: Medizinstudentinnnen waren dabei und stellten wichtige Fragen*  - good concept: medical students were there and asked important questions | *- manche Themen wurden etwas ausschweifend behandelt, wie z.B. am Anfang die Definition der Begriffe  - die Zusammenfassungen zwischendurch weglassen, lieber schriftlich verfassen und bei der Folge verlinken, sodass man sich das am Ende nochmal durchlesen kann bzw. auch als Sicherung, um sich das später nochmal anzuschauen - mehr Zeit für Besprechung der Therapie aufwenden*  - some topics were dealt with in a bit too much detail, e.g. the definition of terms at the beginning.  - leave out the summaries in between, instead write them down with a link in the episode, so that one can read them again at the end or have as a backup to look at again later on  - Spend more time discussing the therapy |
| *Sehr gutes Format! Motiviert mich die Leitlinie anzuschauen.*  Very good format! Motivates me to look at the guideline. |  |
| *Die Fragen der Studierenden*  The student’s questions | *Etwas mehr Basiswissen wiederholen*  Repeat basic knowledge a little more |
| *Der Podcast ist- trotz trockenem Thema- anschaulich und sehr praxisnah!*  The podcast is - despite the dry subject - illustrative and very practical! |  |
| *sehr detailreich, sehr um Hintergrundinformationen und Evidenz für Empfehlungen bemüht, Wiederholung der wichtigsten Fakten & Empfehlungen für erfolgreiches Lernen, hochkarätige ExpertInnen*  Very detailed, great effort to provide background information and evidence for recommendations, repetition of key facts & recommendations for successful learning, top-notch experts | *Die Titel der Folgen machen zwar neugierig, sollten meiner Meinung nach aber das behandelte Thema konkret nennen (auch wenn das viel langweiliger wäre), um es zu erleichtern, bestimmte Passagen erneut anzuhören ohne lange zu suchen, in welcher Folge welches Krankheitsbild behandelt wird.*  The titles of the episodes make you curiosity, but in my opinion they should state specifically the subject treated (even if that might be much more boring) to make it easier to listen to certain passages again without having to search for a long time to find the episode that treats that clinical picture. |
| *Interessant zur Ergänzung regulärer Lehre, besonders nice sind die Fragen aus Studierendenperspektive, das sind die Fragen, die man sich selbst auch stellt.*  Interesting supplement to regular teaching, the questions from the student perspective are especially nice, those are the questions you ask yourself too. | *Bisschen mehr Struktur wäre manchmal nicht verkehrt*  A bit more structure sometimes wouldn’t hurt |
| *Super Podcast, vielen Dank!*  Great podcast, thanks a lot! |  |
| *Inhaltlich sehr schöne und kurzweilige Folge. Vielen Dank für die Mühe!*  In terms of content, very nice and entertaining episode. Thanks for taking the effort! | *- Das Gendern im mündlichen Sprachgebrauch stört und lenkt vom Inhalt ab. Bitte lieber Formulierungen wie "Patientinnen und Patienten" verwenden statt "Patient_innen". - Gerne zusätzlich auch etwas kürzere Folgen für zwischendurch. - Die Zeile in diesem Fragebogen zum Studiensemester funktioniert nicht.*  - The use of gender-sensitive language in oral speech is bothersome and distracts from the content. Instead please use the German feminine and masculine forms instead of the German gender inclusive term.^[[1]](#footnote-2)^  - In addition, please include somewhat shorter episodes for in between  - The line about the study semester in this questionnaire does not work. |
| *Super interessant und exzellent produziert, und moch was dazugelernt :)*  Super interesting and excellently produced, and I learned some new things :) |  |
| *Wirklich gut gemacht, sehr anschaulich, sehr gut verständlich. Deutlich besser gemacht, als die xy Folge.*  Really well done, very clear, very easy to understand. Much better done than the xy episode. | *Trotzdem zu lang.*  But still too long. |
| *Super informativ und gute Tipps für den klinischen Alltag*  Super informative and good tips for everyday clinical practice |  |
| *Super erklärt! Was sonst Trocken und eher unbeliebt ist, ist hier so mühevoll aufarbeitet, dass die Faszination ansteckend ist! Besonders gut finde ich, dass zwischendurch nochmal zusammen gefasst wird und rekapituliert wird.*  Super explained! What is otherwise dry and rather unpopular, is worked up with so much care here that the interest is contagious!  I think it is especially good that in between things are summarized and recapitulated. | *Die Zusammenfassung bzw Key-Aspekte in den Show-notes wären das Non plus Ultra!!*  A summary or key aspects in the show-notes would be the greatest!!! |
| *Zusammenfassungen der Moderatorin/Ärztin, die helfen sehr! Gute Struktur Medizinstudentin aus unterschiedlichen Semestern*  Moderator/physician summaries, they really help!  Good structure  Medical student from various semesters |  |
|  |  |
| *Endlich habe ich wirklich verstanden, was MRSA ist, danke*  Finally, I really understood what MRSA is, thank you | *Vielleicht doch nochmal eine Folge mit einer groben Übersicht der wichtigsten Erreger und der wichtigsten Antibiosen*  Perhaps an episode with a rough overview of the most important pathogens and the most important antibiotic therapies |
| *Didaktisch super!*  Great for learning! |  |
| *Der 2. Teil war wirklich gut und auch interessant.* The 2nd part was really good and interesting too. | *Der 1. Teil war deutlich zu lang und bestand aus den absoluten Basics. Ich finde 1.5h insgesamt sehr sehr lang. Man hätte im 1. Teil vielleicht Zeit sparen können, indem man die Fragen der Studenten weglässt bzw stark verkürzt. Zb, was macht man, wenn jemand eine xy Infektion hat? Es ist mir sehr schwer gefallen, solange zuzuhören und ich mache dann immer etwas nebenbei. Zum einen liegt das sicher daran, dass ich die Grundlagen kenne. Andererseits interessieren mich Infektio Themen sehr. Max 35 min insgesamt, würde ich sagen.*  The 1st part was much too long and consisted of the absolute basics. I find 1.5 hours in total very very long. One could have perhaps saved time in the 1st part by omitting or greatly shortening the questions from the students. For example, what do you do if someone has an xy infection? It was very difficult for me to listen as long as I did. I then always do something else at the same time. On the one hand, this is certainly because I know the basics. However, infection topics do interest me a lot. I would say max 35 min in total. |
| *Sehr gut zum lernen*  Very good for learning | *Zu lang*  Too long |
| *Sehr guter Gast mit exzellenter Expertise!*  Very good guest with excellent expertise! | *Manchmal könnte es etwas kompakter sein*  Sometimes it could be a little more concise |
| *Ich bin Apothekerin in einer Klinik und habe sehr viel bei euch gelernt! Danke*  I am a pharmacist in a clinic and learned a lot from you! Thank you |  |
| *Toller Podcast*  Great Podcast |  |
| *Übersichtlich und reduziert auf das was klinisch relevant ist*  Clear and reduced to what is clinically relevant |  |
| *Hat mir gut gefallen, habe viel gelernt*  I liked it very much, I learned a lot |  |
| *Ich bin total begeistert von den Folgen! Ich habe das Gefühl, durch das strukturierte Abarbeiten von Klinik, Diagnostik und Therapie wirklich wahnsinnig viel zu lernen. Bitte macht auf jeden Fall so weiter! Das Lernen macht so richtig Spaß :)*  I am totally excited about the episodes! I feel like I'm really learning an insane amount by working through the clinical picture, diagnostics and therapy in a structured way. Please keep up the good work! Learning is so much fun :) |  |
|  | *Ich würde mich freuen wenn hin und wieder die Arbeit der technischen AssistenInnen erwähnt werden würde.*  I would be happy if every now and then the work of the technical assistants were mentioned. |
| *Ich bin schon seit 3 Jahren als Assistenzarzt für Nephrologie in Österreich tätig und lerne bei jeder Folge viele Dinge dazu. Teilweise wird auch durch die klinische Praxis anders angelernes und damit aus dem Studium verloren gegangenes Wissen wieder belebt sodass man seine Arbeitsweise verbessern kann. Ich bin generell ein Fan von medizinischen Podcasts, leider gibt es wenige gute aus dem deutschsprachigen Raum und die medizinische Praxis unterscheidet sich teilweise schon beträchtlich von den USA. Dieser Podcast ist auf jeden Fall empfehlenswert!*  I have been working as a nephrology resident in Austria for 3 years now and I learn lots of new things from every episode. In some cases, knowledge that was lost in clinical practice is revived, so that you can improve the way of you work. I am generally a fan of medical podcasts, unfortunately there are very few good ones from German-speaking countries and medical practice is sometimes quite different from the US. This podcast is definitely recommendable! |  |
| *Sehr ausführlich*  Very thorough |  |
| *Super Podcast, sehr angenehme Atmosphäre und unfassbar hilfreich für den Alltag und die Vorbereitung auf mein M3 in Mikrobiologie!*  Super podcast, very pleasant atmosphere and incredibly helpful for everyday life and preparation for my M3 [= third phase of the medical exam] in microbiology! |  |
| *Toll Struktur der Folge Sehr verständlich erklärt Sehr gute*r Dozent*in Die Zusammenfassungen der Moderatorin nach jedem Themenblock sind sehr hilfreich!*  Great structure of the episode  Very understandibly explained  Very good lecturer  The moderator's summaries after each section of the topics are very helpful! |  |
| *Absolut empfehlenswert, auch für erfahrene Kliniker. Sehr gute Zusammenfassung des Themas.*  Absolutely to be recommended, even for experienced clinicians. Very good summary of the topic. |  |
| *Sehr praxisrelevant!*  Very relevant in practice! |  |
| *Sehr gut sind die kurzen Zusammenfassungen!*  The short summaries are very good! | *Die ausführliche Vorstellung irgendwelcher Studentinnen - peinlich.*  Introduction of random students in such detail - embarrassing |
| *Themenauswahl super, sehr lehrreich, gut zuzuhören durch den Interviewcharakter, die Studenten scheuen sich nicht, auch „einfache“ Fragen zu stellen, Zwischenfazits durch die Moderatorin- top.*  The choice of topics was super, very informative, easy to listen to due to the nature of the interview, the students were not afraid to ask "simple" questions, interim conclusions of the moderator – the best. | *Einzig die Länge. Übliche Länge einer Podcastfolge ist ca. 45-60 min. Hat was mit der Aufmerksamkeitsspanne zu tun. Zudem ist es bei zunehmender Länge schwieriger im Vorfeld zu sondieren, welche Themen behandelt werden und ob diese Folge für einen sehr relevant ist oder nicht. Lieber kürzere Folgen mit enger zugeschnittenen Themen, die man den Shownotes/der Beschreibung gut entnehmen kann, als sehr lange Gespräche, wo dies und das und jenes mit angeschnitten wird.*  Only the length. The usual length of a podcast episode is about 45-60 minutes, which has something to do with attention span. Also, as the length increases, it's harder to to get a feeling in advance for what topics are being covered and whether the episode is very relevant for you or not. It's better to have shorter episodes with more narrowly tailored topics that you can read about in the show notes/description than really long conversations where this and that and the other area all touched on. |
| *Mega spannend und kurzweilig zu hören. Freue mich über jede neue Folge! Es ist ein sehr relevantes Thema. Die Aufmachung mit den studentischen Fragestellern und entsprechenden Experten gefällt mir sehr!*  Totally exciting and entertaining to listen to. I look forward to each new episode! It is a very relevant topic. I really like the set-up with the student questioners and different experts! | *Gerne mehr neue Folgen!*  Please more new episodes! |
| *xyz ist ein sehr angenehmer Experte für dieses Podcast Format*  xyz is a very nice expert for this podcast format |  |
| *Sehr gutes Format auch für Berufseinsteiger! Pragmatisch bzw Praxis relevanter Inhalt für den Klinikalltag. Bitte weiter so!*  Very good format for new professionals too! Pragmatic and practice-relevant content for everyday clinical work. Please keep it up! |  |
| *Richtig toller Podcast. Macht weiter so.*  Really great podcast. Keep up the good work. |  |
| *Großartige Folge!*  Great episode! | *Keine Kritik - einfach so weitermachen :-)*  No criticism - just keep it up :-) |
| *Wichtige Thema, wieder kurzweilig aufgearbeitet. Doppelt interessant als Anästhesist und gerade in der Zusatz-WB Infektiologie...*  Important topic, once again entertainingly presented. Doubly interesting as an anesthesiologist who is at the moment doing additional training in infectiology... | *Ggf. Skript der Folge?*  Script of the episode, if possible? |
| *Hervorragend! Ich liebe diesen Podcast*  Outstanding! I love this podcast |  |
| *Kurz, prägnant, verständlich - auch für den Kliniker richtig gut*  Short, concise, understandable - really good for clinicians too |  |
| *Finde es sehr gut dass ihr keine Angst vor längeren Folgen habt. Wichtige Themen, toller Podcast*  I really like that you are not afraid of longer episodes. Important topics, great podcast |  |
| *Tolle Experten, gut finde ich die Zusammenfassung, die Zwischendurch erfolgt. Sehr Praxisrelevant*  Great experts, I like the summary done in between. Very relevant to practice | *Die Folgen sind recht lang. Evtl. weniger Themen in einer Folge.*  The episodes are quite long. Maybe fewer topics in an episode. |
| *Vielen Dank, für euer Engagement!*  Thank you very much for your commitment! |  |
| *Sehr gute Themen und tolle Gäste! Durch die Detailtiefe lerne ich als Assistenzärztin sehr viel!*  Very good topics and great guests! I learn a lot as a resident because of the depth of detail! | *Das Intro finde ich etwas lang, hier könnte man kürzen.*  I find the intro a bit long, you could shorten it here. |
| *Auch für Apothekerinnen sehr interessant*  Very interesting for pharmacists too |  |
| *Super Referentinnen- und Referentenauswahl. Modernes Format zur Infektiologie. DANKESCHÖN!*  Super selection of speakers. Modern format on infectiology. THANK YOU! | *Vorspann und Abspann dürfen deutlich kürzer sein.*  Opening and final credits could be significantly shorter. |
|  | *Die Fragen der Studierenden sind toll, trotzdem wäre manchmal etwas mehr Struktur angenehm. Wenn man nicht ganz im Thema ist, kommt man zum teil etwas schwer rein.*  The questions from the students are great, but sometimes a little more structure would be nice. If you are not completely into the subject, it is sometimes difficult to get into it. |
| *Sehr informativ*  Very informative | *Dieser Podcast ist definitiv nicht nur für Berufsanfänger geeignet, gerade bei diesem Thema sind doch die allermeisten Fachdisziplinen überfordert und ratlos.*  This podcast is definitely not just suitable for newcomers to the profession, with this topic, in particular, the vast majority of disciplines are overwhelmed and at a loss. |
| *Auswahl der Expert*innen wieder sehr gut, vor allem dass das Thema aus zwei Richtungen beleuchtet wurde*  Selection of experts once again very good, especially that the topic was examined from two perspectives |  |
| *Ausführlich und toll. Es ist schön dass Themen nicht abgeblockt werden oder aus zeitlichen Gründen nicht angesprochen werden, sondern dass einfach die Zeit da ist alle Fragen ausführlich zu beantworten.*  Thorough and great. It is nice that topics are not rejected or not discussed due to time constraints, that there is simply time to answer all questions in detail. |  |
| *Super aufgearbeitet, auch für bereits länger tätige Ärzte sehr informativ.*  Done very well, also very informative for clinicians who have been practicing for a while. |  |
| *xyz wartete mit einem großen Überblick auf. Ich liebe es, wenn gelehrte Menschen die Dinge so vereinfachen und auf den Punkt bringen. Format super, bitte macht weiter!*  xyz [expert] came up with a great overview. I love it when educated people simplify things like this and get to the point.  Format great, please keep it up! | *Bitte versucht doch, die Folgen wieder deutlich < 1 Std. zu kriegen. Z.Zt. ufert es doch ziemlich aus.*  Please try to get the episodes down to < 1 hour again. At the moment it's getting pretty out of hand. |
| *Ich höre die Folge, weil ich als Journalistin einen Artikel über das Thema schreibe. Insgesamt sehr interessant und gut verständlich.*  I listened to the episode because I am a journalist writing an article on the subject.  Overall very interesting and easy to understand. | *Manchmal werden Begriffe nicht erklärt (z. B. FEV1) oder Fachwissen schon vorausgesetzt. Dann versteht man die Info insgesamt nicht genau (bzw. muss schnell googeln). Kurze Erläuterung von Fachbegriffen wäre hilfreich. (Ich weiß nicht, ob die Mediziner alle Begriffe schon kennen; aber Medizinstudierenden geht es ja vielleicht ähnlich...)*  Sometimes terms are not explained (e.g. FEV1) or specialist knowledge is assumed.  Then one doesn't exactly understand the information (or you have to google it quickly). A brief explanation of technical terms would be helpful.  (I don't know if the physicians already know all the terms; but medical students might feel the same way...) |
| *Super strukturiert, interessant und kurzweilig!*  Super structured, interesting and entertaining! | *diesmal keine*  none this time |
| *Höre die Folgen in Vorbereitung auf das m2.  Und lerne jedes Mal was dazu :) danke!*  Listen to the episodes in preparation for the m2 [=2nd phase of the medical exam]. And learn something new every time :) thanks! |  |
| *Ganz ganz tolles Format in dieser Folge!! Ich hab die Folge 2 mal gehört und beim zweiten Mal sogar Notizen gemacht. So viel Information in einem Podcast! Ganz toll-vielen Dank und weiter so!!:)*  Really really great format in this episode!! I listened to the episode 2 times and even took notes the second time. So much information in one podcast! Really great - thank you and keep up the good work!! :) |  |
| *Das war eine tolle Übersicht anhand von Fallbeispielen Man könnte wirklich viel mitnehmen*  A great overview based on case studies You can really get a lot out of it |  |
| *Wie immer sehr gut. Insbesondere die Kombination aus theoretischem Wissen und klinischer Anwendung. Die Zusammenfassungen sind sehr hilfreich.*  As always, very good. Especially the combination of theoretical knowledge and clinical application.  The summaries are very helpful. |  |
| *Sehr gute Folge*  Very good episode |  |
| *Super Projekt, wenn ich irgendwo etwas für die Finanzierung vorschlagen könnte, würde ich euch wählen. Ich schreibe nöchste Woche Pharmakologie und ihr habt mir enorm geholfen, gerade die interdisziplinären Anteile finde ich super. Ich hoffe es kann ganz bald weitergehen!*  Super project, if I were able suggest something for funding somewhere, I would choose you. I am writing pharmacology next week and you have helped me enormously, I find the interdisciplinary parts especially super. I hope it can continue very soon! |  |
| *Ihr bringt theoretischen Hintergrund in den Alltag. Das hilft sehr die Rationale zu verdeutlichen.*  You bring theoretical background into everyday life. This helps a lot to make the rationale clearer. |  |
|  | *Gut, Sie haben sich einen Veterinär eingeladen - da verwundert es nicht sehr, dass nicht EIN MAL die Massentierhaltung selbst kritisch in Frage gestellt wird. Zoonosen, Pandemien, Gewässerbelastung und die Tatsache, dass der Konsum von tierischen Produkten extrem ineffizient ist und damit einen nicht unerheblichen Faktor zur Klimakatastrophe beiträgt, werden einfach nicht erwähnt - oder, wenn erwähnt als notwendiges Übel dargestellt.  Bisweilen wird doch im Nebensatz "Tierwohl" genannt - aber Mastbetriebe mit Hühnern, bis zu 30.000 (dreißigtausend!!) Tieren in einer Kolonie, ja das ist konventionelle Tierhaltung - ist halt so(!), muss man auch nix dran ändern, wird ja alles immer teuerer. . . Der Ausdruck "Tierwohl" in diesem Zusammenhang verhöhnt sowohl die ausgebeuteten Lebewesen, als auch die "Endverbraucher", die sich für billige Tierprodukte gerne anlügen lassen.*  *Viehwirte und ihre Veterinäre möchten gerne so gesehen werden, dass sie sich tatsächlich für das "Tierwohl" einsetzen - dies kritisch in Frage zu stellen, wäre eine Aufgabe dieses Themenkomplexes gewesen. Wäre. . . - und nein, ein "Strohschwein" macht es auch nicht besser, das ist Greenwashing und kommt kaum vor. 99% unseres "Nutzviehs" kommt aus der sogenannten konventionellen Haltung.  Ich liebe Ihren Podcast und habe in den anderen Folgen extrem viel gelernt! Aber die derartig unkritische Darstellung dieses Themas war eine herbe Enttäuschung.*  Good, you invited a veterinarian - so it is not very surprising that factory farming itself isn’t critically questioned ONCE. Zoonoses, pandemics, water pollution, and the fact that the consumption of animal products is extremely inefficient and thus contributes not insignificantly to the climate catastrophe are simply not mentioned - or, if mentioned, are presented as a necessary evil.  "Animal welfare" is sometimes mentioned in passing - but fattening farms with chickens, up to 30,000 (thirty thousand!!) animals in a colony, yes that's conventional livestock farming - that's just the way it is(!), you don't have to change anything about it, everything is getting more and more expensive. . . The expression "animal welfare" in this context is a mockery of both the exploited creatures, as well as the "end consumers" who are willing to be lied to in exchange for cheap animal products.  Livestock farmers and their veterinarians like to be seen as actually working for “animal welfare” - critically questioning this would have been a task of this comples of issues. Would have been . . - and no, a "pig raised in straw" doesn't make it any better, that's greenwashing and hardly ever happens. 99% of our “livestock” comes from so-called conventional farming.  I love your podcast and have learned an a great deal from the other episodes! But such uncritical presentation of this topic was a bitter disappointment. |
| *Wie immer super!*  Great as always! |  |
| *Super*  Great | *super*  great |
| *Wirklich top! Ein Aspekt hat mir noch gefehlt : wenn ein anderer Infekt besteht wie zb covid, hat es der staph. Aureus leichter, wird aber nicht oder später erkannt. Was ich sagen will: man kann Läuse und Flöhe haben. Werde den Podcast weiterempfehlen ❤️*  Really the best! One aspect was still missing: if there is another infection like covid, the staph. aureus has it easier, it is not detected or not until later. What I want to say: there can be two reasons for a problem [German figure of speech: "Läuse und Flöhe haben" (to have both lice and fleas)]. Will recommend the podcast ❤️ | *Nur einen! Man findet ihn nicht  Ich habe letztens Podcast über Spotify* *Stichwortsuche gesucht zu Sepsis, Endokarditis und Blutkultur - handling. Und eure Beiträge wurden nicht angezeigt. Das würde ich sogar im Nachhinein noch ändern oder die Suchworte ergänzen.*  Only one! You can't find it  I recently did a keyword search for a podcast on Spotify about sepsis, endocarditis and blood culture handling.  And your contributions didn’t come up.  I would change that later or add to the search words. |
| *die Folge war ganz wunderbar  Freue mich über die „key facts“ fürs Examen!:)*  the episode was really wonderful. Happy about the "key facts" for the exam!:) |  |
|  | Furchtbare Folge. Xyz [Expert*in] schwadroniert über Minuten von Beginn an. Alleine schon die Vorstellung dauert 5 min weil sie das gesamte Spektrum des Fachs erklären will. Nicht auszuhalten die Folge.  Terrible episode. Xyz [expert] rants for minutes from the start. The introduction alone takes 5 minutes because she wants to explain the entire range of the subject. The episode is unbearable. |
| *Ich lerne gerade für meine Prüfung in klinischer Pharmazie im 6. Semester und der Podcast hat mir sehr geholfen einen Überblick über die rationale AB-Therapie zu bekommen.*  I am currently studying for my 6th semester clinical pharmacy exam and the podcast really helped me get an overview of rational AB therapy. |  |
| *Anfangs eher die Basics, im Verlauf der Folge wurden die Themen in guter Tiefe besprochen*  Just the basics at first, as the episode progressed the topics were discussed in good depth |  |
| *xyz ist immer ein Garant für sehr gut strukturierte Vorträge auf höchstem Niveau. Maximaler Lerneffekt. Prima!*  xyz is always a guarantee for very well structured lectures of the highest level. Maximum learning effect. Great! |  |
| *Super Überblick und sehr nette Stimmung (ist für den Lerneffekt schon sehr wichtig).*  Super overview and very nice atmosphere (very important for effective learning). | *Die Antibiotika waren recht schnell runtergerattert, vielleicht ein bisschen ruhiger?*  The antibiotics were rattled off pretty quickly, maybe a little less hectic? |
| *Nicht nur für Medizinstudierende, sondern auch für Pharmaziestudierende und BerufsanfängerInnen im Krankenhaus ein toller Podcast!*  A great podcast not only for medical students, but also for pharmacy students and those starting their careers in hospitals! | *Vorstellungsrunde etwas kürzer halten*  Keep the introductions a little shorter |
|  | *Als Dosisempfehlung pauschal bei Sepsis die Angabe im Beipackzettel zu verdoppeln, halte ich für fahrlässig!!! Literaturempfehlumg??? Ebenso halte ich es für kritisch generell als Initialtherapie für Fokus Bauchraum oder Harnwege Carbapenem einzusetzen. Selbst im Intensiv Setting sollte man sich da mehr Gedanken machen.*  Doubling the amount in the directions for use as a recommended dose in general in the case of sepsis is negligent in my opinion!!! Reference in the literature???  I also consider it critical to generally use carbapenem as initial therapy for a focus on abdomen or urinary tract. Even in the intensive care setting, more thought should be given to this. |
| *Ich höre gerade noch, werde aber jetzt abbrechen, weil das Gestammel und öm öm ömm nicht mehr zu ertragen ist.*  I'm still listening, but I'm going to stop now, because the stammering and öm öm ömm are unbearable. | *Nicht flüssig 6 Punkte aufzählen zu können, wie geht das, wenn man sich für fachkundig hält ömm ömm*  Not being able to enumerate 6 points smoothly, how is that possible if you consider yourself knowledgeable ömm ömm |
| *Bitte unbedingt mehr Folgen, als Berufsanfängerin in der Inneren höre ich die Folgen immer auf dem Weg zur Arbeit und liebe die Mischung aus praxisnah und gründlich Theorievermittelnd!*  Definitely more episodes please, as a young professional in internal medicine I always listen to the episodes on the way to work and love the mix of hands-on and in-depth teaching of theory! |  |

| **6. What other topics would you like to hear about in this podcast format?**  Welche Themen würden Sie noch gern in diesem Podcast-Format anhören? |
| --- |
| *Original German comment*  English translation |
| *„Steckbriefe“ der wichtigsten Antibiotika. Was ist jeweils zu beachten? Wie muss an Nieren oder Leberfunktion angepasst werden?*  "Fact sheets" with the most important antibiotics. What has be considered in each case? How does it have to be adapted to kidney or liver function? |
| *Fallbeispiele mit diagnostisch, aber auch klinisch lehreicher Dynamik*  Case studies with diagnostically but also clinically instructive dynamics |
| *Interpretation von Antibiogramm*  Interpretation of antibiogram |
| *Auch wenn ihr Antibiotika-Podcast macht, fände ich auch Virologie (am relevantesten natürlich Corona) sehr interessant.*  Even though you guys do an antibiotics podcast, I think virology (the most relevant, of course, Corona) would also be very interesting. |
| *Harnwegsinfekte, im Besonderen Interpretation U-Status/Sticks und Kultur.  Bin Anfänger und hier haben viele „nitritpositiven HWI“ und die älteren Kollegen sind sehr großzügig mit Antibiotika.*  Urinary tract infections, in particular interpretation of urinary status and culture.  I am a beginner and here many have "nitrite positive UTI" and the older colleagues are very generous with antibiotics. |
| *Klinische Fälle mit Diskussion über die passen AB-Wahl*  Clinical cases with discussion of appropriate AB choice. |
| *Vllt nochmal eine Folge zum Thema Sepsis und Antibiotische Herangehensweise.*  Perhaps an episode on the topic of sepsis and antibiotic strategies. |
| *MDR-Tuberkulose, Pharmakodynamik und -kinetik der wichtigsten Antibiotikaklassen*  MDR tuberculosis, pharmacodynamics and kinetics of the most important antibiotic classes. |
| *Abklärung und Therapie viraler Infekte - CMV etc.*  Assessment and therapy of viral infections - CMV etc. |
| *Tropenkrankheiten und ihre Behandlung*  Tropical diseases and their treatment |
| *Die Basicfolge Die top 10 antibiosen und wo und wofür sie Anwendung finden*  The basic episode  The top 10 antibiotics and where and for what they are used |
| *Meningitis*  Meningitis |
| *Gastroenterologische Infektionen des tiefen Bauchraums, ohne Clostridien. Z.b. Pankreatitis mit Pseudozysten.*  Gastroenterological infections of the deep abdomen, without clostridia. E.g. pancreatitis with pseudocysts. |
| *Harnwegsinfektionen, Lues, Endokarditis*  Urinary tract infections, syphilis, endocarditis |
| *1.Prothesen- und knocheninfekte  2. Perioperative AB Prophylaxe*  1. prosthesis and bone infections  2. perioperative AB prophylaxis |
| *Pharma*  Pharma |
| *Helicobacter Pylori*  Helicobacter Pylori |
| *Eventuell Infektionen bei geriatrischen Patienten oder Harnwegsinfekte*  Perhaps infections in geriatric patients or urinary tract infections. |
| *Geschlechtskrankheiten Molekulare Diagnostik Tuberkulose - Diagnostik Gastrointestinale Infektionen Antibiotikatherapie*  STDs  Molecular diagnostics  Tuberculosis - Diagnostics  Gastrointestinal infections  Antibiotic therapy |
| *Virale Erkrankungen*  Viral infections |
| *Tuberkulose*  Tuberculosis |
| *Surveillance Tuberkulose*  Surveillance  Tuberculosis |
| *Candidämie / invasive Pilzinfektionen Endokarditis Meningitis*  Candidaemia / invasive fungal infections  Endocarditis  Meningitis |
| *- Wirkungen von Antibiotika (allgemeine Vorstellung der Gruppen und Wirkungsweisen)*  - Effects of antibiotics (general presentation of groups and modes of action). |
| Eine Folge zum Thema „Umgang mit MRGN Keimen“ fände ich spannend  I would find an episode on the topic of "Dealing with multiresistent gramnegative organisms" interesting |
| *Sepsis, neutropenes Fieber / Infektionen bei Neutropenie bzw Immundefizienz*  Sepsis, neutropenic fever / infections in the presence of neutropenia or immunodeficiency |
| *Intensivmedizinische Themen*  Intensive care topics |
| *Intensivmedizinische Themen, multiresistente Keime*  Intensive care topics, multi drug resistent organisms |
| *Sepsis, Knocheninfektionen, Peritonitis, TDM von Antibiotika, Antiinfektiva in der Zahnheilkunde und Gynäkologie*  Sepsis, bone infections, peritonitis, TDM of antibiotics, anti-infectives in dentistry and gynecology. |
| *Als Kinderärztin interessieren mich die pädiatrisch relevanten Krankheitsbilder, z.B. Meningitis, Harnwegsinfekt, Pneumonie*  As a pediatrician, I am interested in relevant clinical pictures for pediatric medicine, e.g. meningitis, urinary tract infection, pneumonia |
| *Das passt vlt nicht in das Konzept, aber mich würde ein Überblick über die relevantesten Resistenzen in der Klinik interessieren (MRGN, MRSA, VRE, EBSL-Bildner,…)*  This may not fit into the concept, but I would be interested in an overview of the most relevant resistances in the hospital (multi drug resistant gramnegatives, MRSA, VRE...). |
| *Streptococcus pyogenes, Tollwut*  Streptococcus pyogenes, rabies |
| *Infektionen bei Diabetes/ Risiko*  Infections in diabetes/ risks |
| *Gern mehr Pharmakologie:))*  More pharmacology would be nice:)) |
| *Alles zu Pharmakologie, gerne mit den tollen Expert*innen auch zu weiteren (nicht-antibiotika-assoziierten) Themen!*  Everything about pharmacology, with the great experts on other (non-antibiotic-associated) topics too! |
| *Antibiotika in der Sepsis, wie dosiere ich, wann muss ich am eine Rotation denken, wie sollte ich rotieren bei... Von der empirischen Initialtherapie bis zur Kinetik bei alten, kranken und organgeschädigten Patienten.*  Antibiotics in sepsis, how do I dose, when do I need to think about rotation, how should I rotate with... From initial empirical therapy to kinetics in elderly, sick, and orally compromised patients. |
| *Freu mich über examensrelevante Themen, da ich gerade in der M2 Vorbereitung bin.*  Happy to hear about exam relevant topics since I am in M2 [= second phase of the medical exam] preparation right now. |
| *Das kritisch kranke Kind erkennen, also ein Beitrag für Kinderärzte.*  Recognizing a critically ill child, on other words, an episode for pediatricians. |
| *Meningitis*  Meningitis |
| *Meningitis*  Meningitis |
| *Vorgehen in der Schwangerschaft/ bei spezifischen Schwangerschaftsinfektionen*  Procedures during pregnancy/ for specific pregnancy-related infections |
| *Sehr gerne das allgemeine Thema Antibiotic-Stewardship, ich bin nicht 100% Sicher ob das über die Folgen aufgeteilt war aber die Grundprinzipien warum wir konkret Antibiotika reduzieren und spezifizieren wollen finde ich sehr wichtig. Auf Station habe ich als Student manchmal das Problem nur das Deeskalationsargument bringen zu können, ohne fundiert über die konkreten Mechanismen der Resistenzbildung argumentieren zu können. Würde mich sehr freuen :-)*  I like the general topic of antibiotic stewardship, I'm not 100% sure if that was divided up over the episodes but I find the basic principles why we want to specifically reduce and specify antibiotics very important. On ward as a student I sometimes have the problem of only being able to bring the de-escalation argument without being able to make a sound argument about the specific mechanisms of resistance formation.  That would be very nice :-) |
| *Alloimmunthrombozytopenie Z.B.*  Alloimmune thrombocytopenia  E.G. |
| *Infekt unklaren Fokus (inkl Therapie Eskalation bei fehlenden ansprechen auf Erstlinie > gibts was besseres als den "klassischen" Verlauf mit sequentiell ampicillin/sulbactam, pip/taz und meropenem?)*  Infections of unknown focus (incl. therapy escalation in case of no response to first line > is there anything better than the "classic" course with sequential ampicillin/sulbactam, pip/taz and meropenem)? |

**7. How old are you?**

*Wie alt sind Sie?*

|  | Number | | Percent |  |
| --- | --- | --- | --- | --- |
| ns | | 3 | 2.2 | |
| 20 to 29 | | 54 | 40.0 | |
| 30 to 39 | | 36 | 26.7 | |
| 40 to 49 | | 30 | 22.2 | |
| 50 to 59 | | 10 | 7.4 | |
| 60plus | | 2 | 1.5 | |
| Total | | 135 | 100.0 | |

**8. Your Sex:**

*Ihr Geschlecht ist:*

|  | Number | | Percent |  |
| --- | --- | --- | --- | --- |
| ns | | 1 | 0.7 | |
| male | | 52 | 38.5 | |
| female | | 82 | 60.7 | |
| Total | | 135 | 100.0 | |

**9. Are you a medical student?**

*Sind Sie Medizinstudent/in?*

| **Yes: n = 42 = 31.1% of all** | **No: n = 89 = 69.5% of all** |
| --- | --- |
| **9.1 What is your current semester of study?**  *Was ist Ihr aktuelles Studiensemester?*   \| Semester \| Number \| Percent of medical students \| \| --- \| --- \| --- \| \| 1 \| 6 \| 14.3 \| \| 3 \| 1 \| 2.4 \| \| 4 \| 1 \| 2.4 \| \| 5 \| 6 \| 14.3 \| \| 6 \| 3 \| 7.1 \| \| 7 \| 1 \| 2.4 \| \| 8 \| 4 \| 9.5 \| \| 9 \| 12 \| 28.6 \| \| ns \| 8 \| 19.0 \| | **9.1 What do you do otherwise?**  *Was machen Sie sonst?*   \| Profession \| Number \| Percent of non-students \| \| --- \| --- \| --- \| \| Resident \| 35 \| 37.6 \| \| Specialist \| 34 \| 36.6 \| \| Other \| 21 \| 22.6 \| \| ns \| 3 \| 3.2 \|  \| Free text specification in "other”  *German original* / Englisch Translation \| Number \| \| --- \| --- \| \| *Apotheke* / Pharmacy \| 1 \| \| *Apotheker*in* / Pharmacist \| 4 \| \| *Hebamme* / Midwife \| 1 \| \| *Krankenhausapotheker*in* / Hospital pharmacist \| 5 \| \| *klinische Pharmazeutin* / Clinical pharmacist \| 1 \| \| *Lehrerin an einer MTA-Schule* / Teacher at a school for medical-technical assistants \| 1 \| \| *Linguistin* / Linguist \| 1 \| \| *Medizinisch technische Assistentin* / medical-technical assistant \| 1 \| \| Mikrobiologie / microbiology \| 1 \| \| *MTLA* / medical-technical laboratory assistant \| 1 \| \| *Pharmaziestudentin* / student pharmacist \| 1 \| \| *Sozialpädagogin* / Social education worker \| 1 \| \| *Wissenschaftsjournalistin* / Science journalist \| 1 \| \| ns \| 1 \| |
| **9.2 Where do you study?**  *Wo studieren Sie?*   \| City/University \| Number \| Percent \| \| --- \| --- \| --- \| \| Berlin \| 9 \| 21.4 \| \| Bochum \| 1 \| 2.4 \| \| Dresden \| 1 \| 2.4 \| \| Düsseldorf \| 1 \| 2.4 \| \| Erlangen \| 1 \| 2.4 \| \| Freiburg \| 2 \| 4.8 \| \| Göttingen \| 3 \| 7.1 \| \| Greifswald \| 1 \| 2.4 \| \| Heidelberg \| 2 \| 4.8 \| \| Homburg-Saar (Universität des Saarlandes) \| 1 \| 2.4 \| \| Jena \| 2 \| 4.8 \| \| Kiel \| 1 \| 2.4 \| \| Lübeck \| 2 \| 4.8 \| \| Mainz \| 1 \| 2.4 \| \| Munich LMU \| 1 \| 2.4 \| \| Nuremberg (Paracelsus Medizinische Privatuniversität) \| 1 \| 2.4 \| \| Other \| 1 \| 2.4 \| \| Tübingen \| 5 \| 11.9 \| \| Würzburg \| 2 \| 4.8 \| \| Vienna \| 1 \| 2.4 \| \| ns \| 2 \| 4.8 \| |  |

**Univariate analysis**

Grade, knowledge gain rating, length rating, praise and criticism by age group.

| **Age - grouped** | | **Not specified** | **20 to 29** | **30 to 39** | **40 to 49** | **50 to 59** | **60 plus** | **p (Chi square test)** |
| --- | --- | --- | --- | --- | --- | --- | --- | --- |
| **Grade = 1** | n (% of age group) | 0 (0) | 43  (79.6) | 31  (86.1) | 25  (83.3) | 7  (70.0) | 1  (50) | 0.01 |
| **Knowledge gain = yes** |  | 1 (33.3) | 51 (94.4) | 31 (86.1) | 25 (83.3) | 10 (100) | 0 (0) | <0.01 |
| **Length = ok** |  | 0 (0) | 48 (88.9) | 31 (86.1) | 24 (80.0) | 9 (90.0) | 1 (50) | <0.01 |
| **Praise** |  | 1 (33.3) | 36 (66.7) | 19 (52.8) | 13 (43.3) | 6 (60.0) | 1 (50) | 0.38 |
| **Criticism** |  | 1 (33.3) | 11 (20.4) | 13 (36.1) | 9 (30.0) | 1 (10.0) | 1 (50) | 0.42 |

Grade, knowledge gain rating, length rating, praise and criticism by sex.

| **Sex - binary** | | **Female** | **Male or not specified** | **p (two-sided Fisher’s Exact)** |
| --- | --- | --- | --- | --- |
| **Grade = 1** | n  (% of sex) | 64 (78.0) | 43 (81.1) | 0.83 |
| **Knowledge gain = yes** |  | 71 (86.6) | 47 (88.7) | 0.80 |
| **Length = ok** |  | 67 (81.7) | 46 (86.8) | 0.48 |
| **Praise** |  | 45 (54.9) | 31 (58.5) | 0.73 |
| **Criticism** |  | 23 (28.0) | 13 (24.5) | 0.69 |

Grade, knowledge gain rating, length rating, praise and criticism by student status.

| **Student status - binary** | | **Medical student** | **other** | **p (two-sided Fisher’s Exact)** |
| --- | --- | --- | --- | --- |
| **Grade = 1** | n (% of student status) | 34 (81.0) | 73 (78.5) | 0.82 |
| **Knowledge gain = yes** |  | 41 (97.6) | 77 (82.8) | 0.02 |
| **Length = ok** |  | 39 (92.9) | 74 (79.6) | 0.08 |
| **Praise** |  | 26 (61.9) | 50 (53.8) | 0.46 |
| **Criticism** |  | 11 (26.2) | 25 (26.9) | 1.00 |

1. Since German gender-inclusive formulations cannot be translated one-to-one into English, only a general translation of the statement can be provided here. [↑](#footnote-ref-2)
