# Supplementary material for: Teaching prudent antibiotic use on the go: a descriptive report on development, utilization and listener satisfaction of an educational podcast format for medical students and young professionals
Source: Antimicrob Resist Infect Control. 2024 May 11;13:50. doi: 10.1186/s13756-024-01402-8 (PMC11088774; doi:10.1186/s13756-024-01402-8)
Supplement: Supplementary file 2 — Supplementary Material 2 [file 13756_2024_1402_MOESM2_ESM.pdf]

Downloads & Streams

Datum
2021-12-20 - 2023-03-09

DOWNLOADS & STREAMS
für den gewählten Zeitraum

38.829

Die Daten werden derzeit monatsgenau angezeigt.

CSV Export

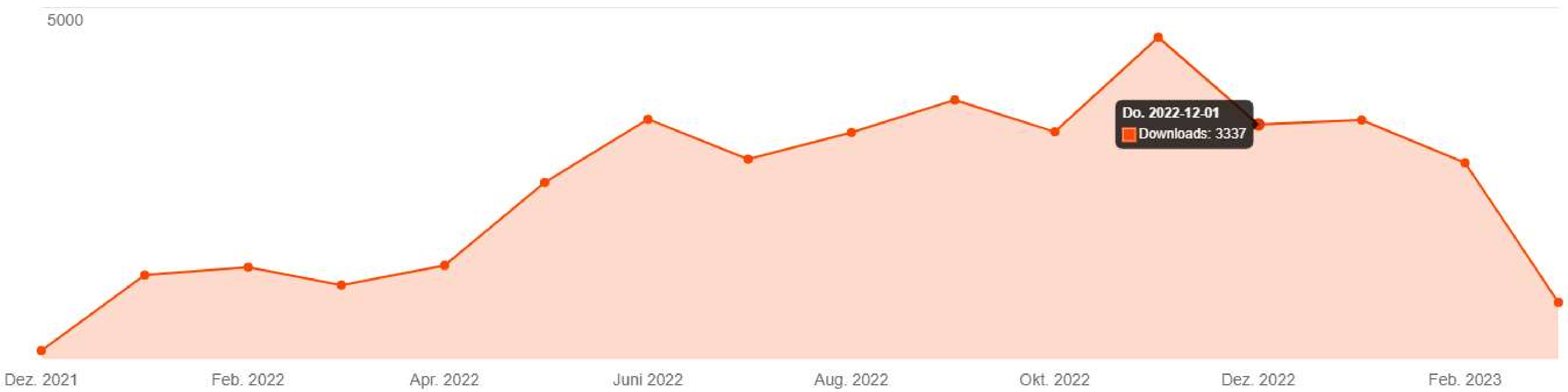

Top 5 Episoden

im Zeitraum von 20. Dezember 2021 bis 10. März 2023

#	Episodentitel	Veröffentlicht am	Anzahl
2	#1 - Von der Hausarztpraxis bis zur ITS	21. Dezember 2021 19:55	5.354
3	#2 - Ist das wirklich ein Harnwegsinfekt?	4. Januar 2022 16:00	3.966
4	#3 - Was wir von den Beduinen lernen können	18. Januar 2022 09:00	2.531
5	#4 - Von Slacklines und anderen Gefahren	1. Februar 2022 07:00	2.245
15	#12 - PharmakoLogisch!	9. August 2022 06:05	2.000

zum Episodenvergleich Alle Episoden

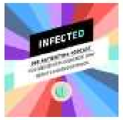

Episodenübersicht

InfectEd: der Antibiotika-Podcast

› InfectEd: der Antibiotika-Podcast

Episodenübersicht

zum Episodenvergleich

Datum
2021-12-17 - 2023-03-09

Suchen nach...

CSV Export

#	Episodentitel	Downloads & Streams	Veröffentlicht am
21	#19 - Schwindsucht	1.596	13. Dezember 2022 06:25
22	#18 - Jede Impfung zählt!	1.174	15. November 2022 06:05
20	#17 - Krank wie noch nie!	1.303	1. November 2022 11:15
19	#16 - Schubladen und das Dazwischen	1.194	18. Oktober 2022 06:00
18	#15 B - ORENUC	1.154	21. September 2022 06:00
17	#15 A - Es brennt!	1.142	20. September 2022 06:00
16	#14 - Woran starb Gustav Mahler?	1.309	6. September 2022 06:05
14	#13 - Über den Tellerrand	1.073	23. August 2022 06:30
15	#12 - PharmakoLogisch!	2.000	9. August 2022 06:05
13	#11 - Morbus Marlboro	1.399	26. Juli 2022 07:25
12	#10 - Sind es vielleicht Pilze?	1.520	12. Juli 2022 08:54
11	#9 - Ausruh'n, Ibu oder doch ein Antibiotikum?	1.700	21. Juni 2022 14:24
10	#8 - PAPperlapapp	1.370	31. Mai 2022 11:49
9	#7 - Late Diagnosis	1.394	3. Mai 2022 20:52
8	#6 - Blut, Urin, Eiter	1.985	29. März 2022 14:03
7	Zwischeninfo	628	15. März 2022 07:00
6	#5 - Primum nihil nocere!	1.766	15. Februar 2022 07:00
5	#4 - Von Slacklines und anderen Gefahren	2.245	1. Februar 2022 07:00
4	#3 - Was wir von den Beduinen lernen können	2.531	18. Januar 2022 09:00
3	#2 - Ist das wirklich ein Harnwegsinfekt?	3.966	4. Januar 2022 16:00
2	#1 - Von der Hausarztpraxis bis zur ITS	5.354	21. Dezember 2021 19:55
1	Trailer	1.027	20. Dezember 2021 17:45

Download-Quellen

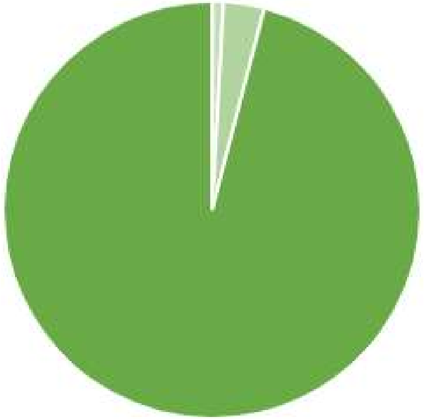

Suchen nach...

CSV Export

Download-Quelle	Anzahl
feed	37.258
webplayer	1.230
unknown	319
social	17
webplayer-download	5

Plattformen

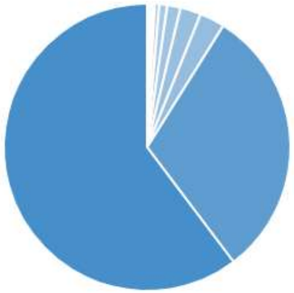

Suchen nach... | CSV Export

Plattform	Anzahl
iPhone	22.093
Android	11.281
Windows	1.037
Unbekannt	855
Mac	552

Plattform	Anzahl
iPad	326
Apple Watch	188
Amazon Echo	78
Apple Device	76
GNU/Linux	66

Sonos	38
HomePod	9
Apple TV	9
ue43mu6179	5
Fedora	5
webOS	4
TPM191E	3
Chromecast	3
iPod touch	2
gq65ls03tauxzg	2
CentOS	2
un55nu7300	2
TPM171E	1
Gentoo	1
ue65ku6400	1
Red Hat	1
Alexa-capable device	1
gq75qn90aatxzg	1
SUSE	1
OpenBSD	1
Fire TV Stick Lite (Gen 1)	1

Apps

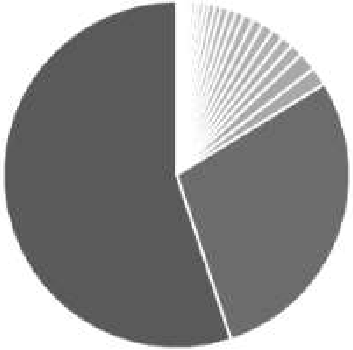

Suchen nach...

CSV Export

App	Anzahl
Spotify	21.389
Apple Podcasts	11.137
Google Podcasts	672
Overcast	670
unknown	470

Zeige 1 bis 5 von 47 Einträgen

5

1 2 3 ... 10

Chrome Mobile	427
Podcast Addict	420
Firefox	417
PocketCasts	379
Mobile Safari	373
Chrome	298
CastBox	281
AntennaPod	279
Deezer	228
Microsoft Edge	223
Safari	170
Amazon Music	167
Samsung Browser	140
Android Browser	134
Downcast	102
Podimo	86
Amazon Alexa	79
Ecosia	47
Firefox Mobile	41
Sonos	38

iTunes	24
Podcast Republic	23
Podbean	22
iCatcher!	13
Opera	12
Facebook in-app browser	12
Chrome Mobile iOS	8
Instacast	7
Firefox Mobile iOS	6
Opera Mobile	5
Google Search App	5
DuckDuckGo Privacy Browser	5
Podcaster	4
Instagram in-app browser	4
LinkedIn	2
Radio.net	2
Yandex Browser	2
Headless Chrome	2
Mobile Silk	1
Procast	1
Chrome Webview	1
Internet Explorer	1

Apple Podcasts Vorschau

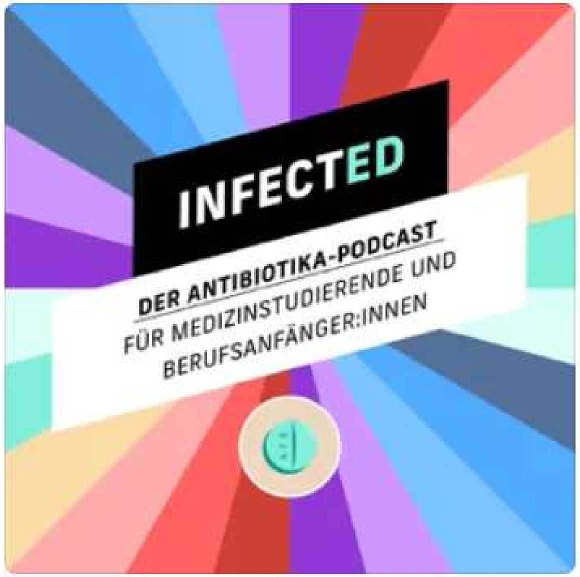

22 Folgen

InfectEd ist eine Charité-Produktion in Zusammenarbeit mit dem Institut für Infektionsmedizin und Krankenhaushygiene des Universitätsklinikums Jena und dem Institut für Hygiene und Mikrobiologie der Universität mehr

InfectEd: der Antibiotika-Podcast

Charité - Institut für Hygiene; UKJ - Institut für Infektionsmedizin

Gesundheit und Fitness

★★★★★ 4,9 • 21 Bewertungen

Anhören in Apple Podcasts ↗

13. DEZ. 2022

#19 - Schwindsucht

Tuberkulose mit Dr. med. Brit Häcker und Dr. med. Ralf Otto-Knapp

▶ WIEDERGABE 1 Std. 46 Min.

15. NOV. 2022

#18 - Jede Impfung zählt!

Impfen und Antibiotikaresistenzen mit Dr. med. Miriam Wiese-Posselt

▶ WIEDERGABE 2 Std 2 Min.

1. NOV. 2022

#17 - Krank wie noch nie!

Sepsis mit PD Dr. med. Matthias Gründling

▶ WIEDERGABE 1 Std. 18 Min.

InfectEd: der Antibiotika-Podcast

Übersicht Trends Folgen Leistung

Gesamtzeitraum

FOLLOWER:INNEN	HÖRER:INNEN	TREUE HÖRER:INNEN	WIEDERGABEN
513	783	518	24,7K

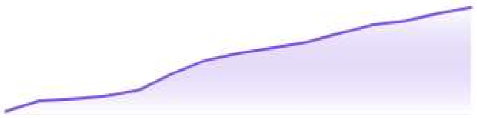

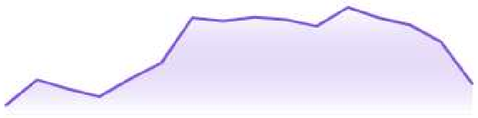

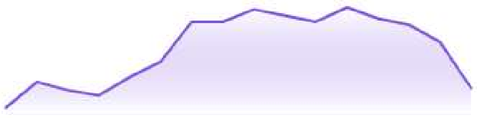

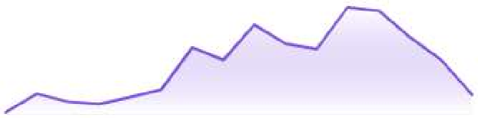

Neue Follower:innen

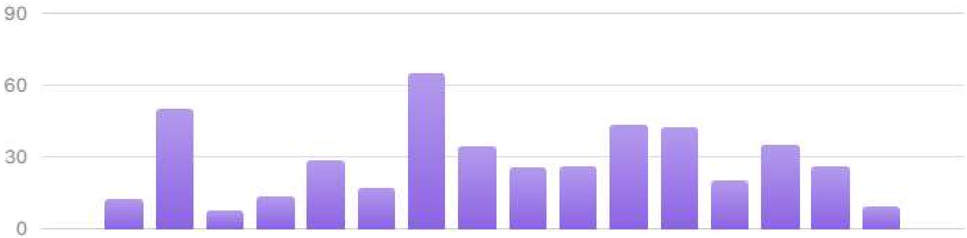

Gehörte Zeit

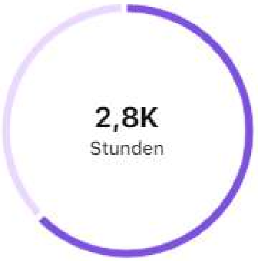

63 % 1,8K Follower:innen

37 % 1,0K Keine Follower:innen

Top-Länder/Top-Regionen Hörer:innen

Alle anzeigen

Deutschland	728
Österreich	30
Schweiz	16
Italien	6

Top-Städte Hörer:innen

Alle anzeigen

Munich	203
Berlin	125
Bonn	123
Dortmund	55
Hamburg	45

InfectEd: der Antibiotika-Podcast

Übersicht Trends Folgen Leistung

Gesamtzeitraum

Folgen (22)

Suchen Alle Folgen

NAME	VERÖFFENTLICHUNGSDATUM	LAUFZEIT	HÖRER:INNEN	TREUE HÖRER:INNEN	WIEDERGABEN	DURCHSCHNITTLICHE NUTZUNG
#19 - Schwindsucht	13. Dez. 2022	1 hr 46 mins	147	88	977	50 %
#18 - Jede Impfung zählt!	15. Nov. 2022	2 hrs 3 mins	111	51	600	36 %
#17 - Krank wie noch nie!	1. Nov. 2022	1 hr 19 mins	118	76	871	64 %
#16 - Schubladen und das Dazwischen	18. Okt. 2022	1 hr 27 mins	114	72	1 168	67 %
#15 B - ORENUC	21. Sep. 2022	50 mins	114	56	582	67 %
#15 A - Es brennt!	20. Sep. 2022	1 hr 14 mins	99	73	765	83 %
#14 - Woran starb Gustav Mahler?	6. Sep. 2022	1 hr 18 mins	114	71	959	73 %
#13 - Über den Tellerrand	23. Aug. 2022	1 hr 13 mins	99	42	437	48 %
#12 - PharmakoLogisch!	9. Aug. 2022	1 hr 40 mins	168	118	1 807	72 %
#11 - Morbus Marlboro	26. Juli 2022	1 hr 8 mins	137	84	872	74 %
#10 - Sind es vielleicht Pilze?	12. Juli 2022	1 hr 51 mins	138	97	1 449	70 %
#9 - Ausruh'n, Ibu oder doch ein Antibiotikum?	21. Juni 2022	1 hr 43 mins	149	84	1 200	59 %
#8 - PAPperlapapp	31. Mai 2022	1 hr 12 mins	119	76	936	70 %

InfectEd: der Antibiotika-Podcast

Übersicht Trends Folgen Leistung

Gesamtzeitraum

#10 - Sind es vielleicht Pilze?	12. Juli 2022	1 hr 51 mins	138	97	1 449	70 %
#9 - Ausruh'n, Ibu oder doch ein Antibiotikum?	21. Juni 2022	1 hr 43 mins	149	84	1 200	59 %
#8 - PAPperlapapp	31. Mai 2022	1 hr 12 mins	119	76	936	70 %
#7 - Late Diagnosis	3. Mai 2022	1 hr 35 mins	99	64	839	68 %
#6 - Blut, Urin, Eiter	29. März 2022	1 hr 41 mins	140	89	1 228	63 %
Zwischeninfo	15. März 2022	2 mins	42	29	51	65 %
#5 - Primum nihil nocere!	15. Feb. 2022	1 hr 48 mins	106	65	690	53 %
#4 - Von Slacklines und anderen Gefahren	1. Feb. 2022	1 hr 40 mins	124	96	1 567	74 %
#3 - Was wir von den Beduinen lernen können	18. Jan. 2022	1 hr 34 mins	142	104	1 519	71 %
#2 - Ist das wirklich ein Harnwegsinfekt?	4. Jan. 2022	1 hr 33 mins	219	145	2 101	67 %
#1 - Von der Hausarztpraxis bis zur ITS	21. Dez. 2021	1 hr 35 mins	303	218	3 924	79 %
Trailer	20. Dez. 2021	3 mins	118	82	169	75 %

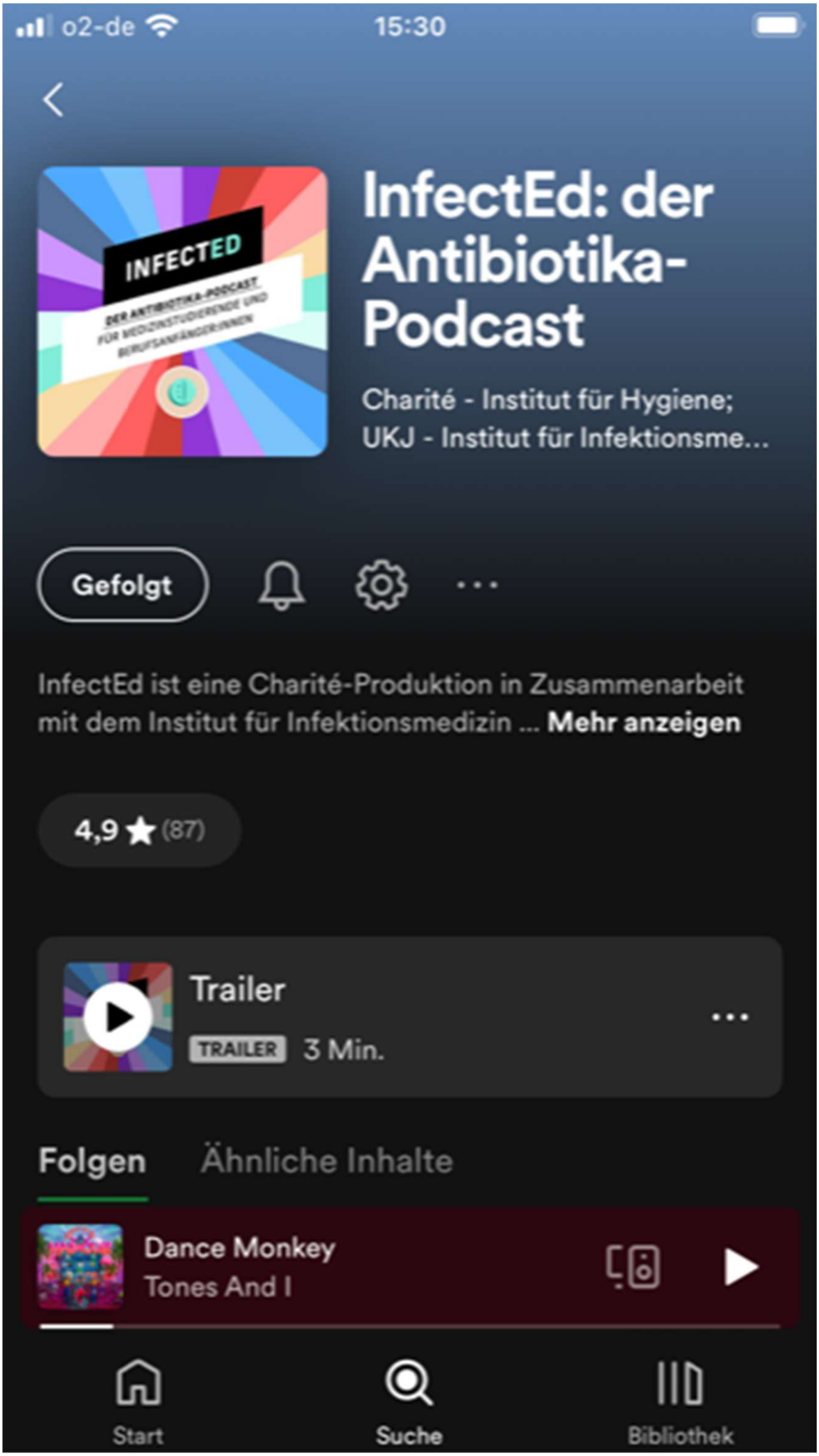
o2-de
15:30
InfectEd: der Antibiotika-Podcast
Charité - Institut für Hygiene;
UKJ - Institut für Infektionsme...
INFECTED
Gefolgt
InfectEd ist eine Charité-Produktion in Zusammenarbeit mit dem Institut für Infektionsmedizin ... Mehr anzeigen
4,9 ★ (87)
Trailer
TRAILER 3 Min.
Folgen
Ähnliche Inhalte
Dance Monkey
Tones And I
Start
Suche
Bibliothek
